# Assessment of Sarcopenia Among Community-Dwelling At-Risk Frail Adults Aged 65 Years and Older Who Received Multidomain Lifestyle Interventions: A Secondary Analysis of a Randomized Clinical Trial

**DOI:** 10.1001/jamanetworkopen.2019.13346

**Published:** 2019-10-16

**Authors:** Yanxia Lu, Mathew Niti, Keng Bee Yap, Crystal Tze Ying Tan, Ma Shwe Zin Nyunt, Liang Feng, Boon Yeow Tan, Gribson Chan, Sue Anne Khoo, Sue Mei Chan, Philip Yap, Anis Larbi, Tze Pin Ng

**Affiliations:** 1 Singapore Immunology Network, Biology of Ageing Laboratory, Agency for Science Technology and Research, Biopolis, Singapore; 2 Performance and Technology Assessment Department, Ministry of Health, Singapore; 3 Geriatric Medicine and Palliative Medicine Department, Ng Teng Fong General Hospital, Singapore; 4 Gerontology Research Programme, Yong Loo Lin School of Medicine, Department of Psychological Medicine, National University Health System, National University of Singapore, Singapore; 5 Medical Services Department, St Luke’s Hospital, Singapore; 6 Rehabilitation Services Division, St Luke’s Hospital, Singapore; 7 Psychological Medicine Department, Khoo Teck Puat Hospital, Singapore; 8 Nutrition and Dietetics Department, Khoo Teck Puat Hospital, Singapore; 9 Geriatric Medicine Department, Khoo Teck Puat Hospital, Singapore; 10 Department of Biology, Faculty of Sciences, University Tunis El Manar, Tunis, Tunisia; 11 Geriatrics Division, Department of Medicine, Research Center on Aging, University of Sherbrooke, Sherbrooke, Quebec, Canada

## Abstract

**Importance:**

There is little understanding of the outcomes associated with active lifestyle interventions for sarcopenia among older persons.

**Objective:**

To determine the association of 6-month multidomain lifestyle interventions (physical exercise, nutritional enhancement, cognitive training, combined treatment, and standard care) with change in sarcopenia status and physical function among adults 65 years and older.

**Design, Setting, and Participants:**

Post hoc secondary analysis of a parallel-group randomized clinical trial conducted from September 1, 2012, to September 1, 2014, at community centers providing services to elderly individuals in Singapore. Participants included a subsample of 92 community-dwelling prefrail or frail older persons with sarcopenia aged 65 years and older. Data were analyzed from June 1, 2017, to January 1, 2018.

**Interventions:**

The 5 intervention groups were a 6-month duration of physical exercise that included resistance and balance training, nutritional enhancement with a commercial oral nutrition supplement formula, cognitive training, a combination of the preceding 3 interventions, and standard care (control).

**Main Outcomes and Measures:**

Primary outcomes were changes in sarcopenia status and its components, appendicular skeletal muscle index (ASMI), knee extension strength (KES), and gait speed (GS) at 3 months and 6 months following the intervention. Sarcopenia was defined as the presence of both low ASMI and low KES and/or GS.

**Results:**

In 92 participants with sarcopenia, the mean (SD) age was 70.0 (4.7) years and 59 (64.1%) were female. Seventy-eight participants received active interventions and 14 received standard care. Of 92 total participants, the number who remained sarcopenic was reduced to 48 (of 73) after 3 months and 51 (of 75) after 6 months of intervention, indicating that 25 of 92 participants (27.2%) experienced sarcopenia reduction at 3 months and 24 of 92 (26.1%) had sarcopenia reduction at 6 months. Low KES was present in 88 of 92 patients (95.6%), and low GS in 30 of 92 patients (32.6%) at baseline. Among the components of sarcopenia, GS had the greatest change associated with active interventions, with 22 of 30 participants (73.3%) free of low GS at 6 months; in comparison, 17 of 88 participants (19.3%) were free of low KES at 6 months and 7 of 92 participants (7.6%) were free of low ASMI at 6 months. Men experienced greater reduction in sarcopenia than women (χ^2^ = 5.925; *P* = .02), as did those with younger age (*t* = −2.078; *P* = .04) or higher ASMI (mean [SD] ASMI, 5.74 [0.77] vs 5.14 [0.77] kg/m^2^; *P* = .002). Participants in the active intervention group experienced statistically significant decreases in sarcopenia score and its components at 3 months and 6 months from baseline (*F* = 14.138; *P* < .001), but the intervention was not associated with significant differences in ASMI, KES, and GS vs standard care.

**Conclusions and Relevance:**

This study suggests that older persons with sarcopenia are responsive to the effects of multidomain lifestyle interventions. Sarcopenia reduction was most pronounced through improved gait speed, and occurred more among those who were male, were younger, or had greater muscle mass.

## Key Points

**Question:**

How is an active lifestyle intervention associated with improvement in muscle mass and function among frail older persons with sarcopenia?

**Findings:**

In this secondary analysis of a randomized clinical trial of physical, nutritional, cognitive, or combined interventions among 92 community dwelling at-risk frail adults aged 65 years and older with sarcopenia, the intervention was associated with a significant reduction in sarcopenia and improved muscle mass and strength and gait speed at 3 months and 6 months. Sarcopenia reversal was more likely to happen in men, younger individuals, and those with higher baseline lean muscle mass.

**Meaning:**

These findings suggest that multidomain lifestyle interventions may be effective in reversing sarcopenia and improving muscle mass and function in community-dwelling at-risk frail older adults.

## Teaser

This secondary analysis of a randomized clinical trial examines the association of a 6-month multidomain active lifestyle intervention with sarcopenia among elderly participants in Singapore.

## Introduction

Sarcopenia is a hallmark of the aging process involving the accelerated loss of skeletal muscle mass, strength, and function.^1^ Authors have pointed out that “there is probably no decline in structure and function more dramatic than the decline in lean body mass or muscle mass over the decades of life.”^2^ Sarcopenia is associated with multiple adverse outcomes, such as falls, multimorbidity, impaired quality of life, disability, and mortality.^3,4^ Developing effective interventions for sarcopenia is vital for reducing the disease burden and increasing the healthy life span of the elderly population.^5^

There is a current consensus that sarcopenia is potentially reversible.^6^ The average nutritional consumption of elderly persons with sarcopenia falls below the Recommended Dietary Allowances for micronutrients.^7^ Studies in humans^8,9,10^ suggest that physical inactivity and anabolic resistance (a blunted synthetic response to protein and exercise) are primary drivers of muscle mass loss in the aging process. Thus, interventions designed to detect and prevent or delay the progression of sarcopenia by targeting primary causes such as inactivity and malnutrition^7,8,9,11^ can potentially improve the quantity and quality of skeletal muscles.^12,13^ There are few studies that have assessed the associations of physical exercise and nutritional intervention with sarcopenia reduction.^14,15,16,17,18,19^ Some studies^15,16^ suggest that physical exercise may be beneficial in improving muscle mass, strength, and gait speed (GS) in elderly people with sarcopenia. There are mixed results regarding the association of nutritional intervention for enhancing muscle strength.^14,15,16,17,18^ However, most studies^17,19^ defined sarcopenia based on the sole criterion of low skeletal muscle, and only 1 Japanese study^15^ additionally involved muscle strength and function by the recent consensus criteria of sarcopenia. Furthermore, the results were generated from relatively short (3- to 4-month) interventions, and it is not known whether the possible benefits of interventions persist over longer durations. Cognitive training is also found to maintain and improve GS and balance in the elderly,^20,21^ although its effects on sarcopenia have not been reported yet. These studies were graded as very low–quality to low-quality trials conducted in heterogeneous populations with relatively short intervention durations and yielded mixed results.^14^ There is still little understanding of the associations of active lifestyle interventions among elderly individuals with reducing sarcopenia and its component muscle mass and function.

We previously reported a randomized clinical trial^22^ of 6-month parallel group multidomain lifestyle interventions (physical exercise, nutritional enrichment, and cognitive training singly and in combination vs standard care control) among prefrail and frail older adults living in the community. In that Frailty Intervention Trial (FIT) in Singapore, sarcopenia was assessed. In this article, we report observations of the associations between interventions and changes in sarcopenia status and component muscle mass and function among participants with sarcopenia at 3 months and 6 months.

## Methods

### Study Design and Participants

**Figure 1.**
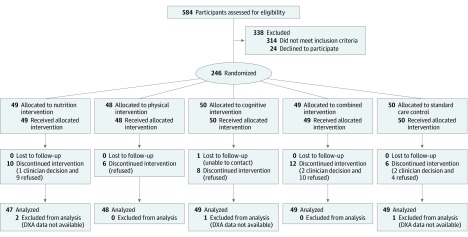
CONSORT Flow Diagram of Frailty Intervention Trial Participant Enrollment and Study Design Participants were included in analysis based on intention to treat. DXA indicates dual-energy x-ray absorptiometry.

Details of the Singapore FIT study have been described in a previous publication^22^ and are briefly summarized in Figure 1. The FIT is a parallel-group randomized clinical trial of community-dwelling older persons who were screened between October 2009 and August 2012 for the physical frailty phenotype using 5 criteria from the work of Fried et al^23^: (1) unintentional weight loss, (2) slow walking speed, (3) weakness, (4) self-reported exhaustion, and (5) low physical activity and determined to be either prefrail (score of 1-2) or frail (score of 3-5) for trial entry. Two-hundred forty-six eligible participants (aged ≥65 years, able to ambulate without personal assistance, and living at home) were randomized to receive one of five 24-week interventions: physical exercise, nutritional enrichment, cognitive training, combined intervention, or standard care. Participants were excluded from the study if they had cognitive impairment (Mini-Mental State Examination^24^ score ≤23) or severe audiovisual impairment, degenerative neurologic disease, major depression, terminal disease with life expectancy 1 year or less, or participation in other interventional studies. This study is a secondary analysis reported in line with the Consolidated Standards of Reporting Trials (CONSORT) reporting guideline based on the interventions completed in September 2014. The study was approved by the National Health Group Domain Specific Review Board of Singapore, and all participants provided written informed consent.

### Sarcopenia Measurement

Sarcopenia, which is the primary outcome of this study, was determined based on appendicular lean mass, lower limb strength, and GS according to the Asian Working Group for Sarcopenia criteria^25^ released in 2014.

Appendicular lean body mass was measured by dual-energy x-ray absorptiometry scan with a bone densitometer (Hologic). Scans were performed in accordance with the manufacturer’s protocol in the Department of Diagnostic Radiology, National University Hospital of Singapore. Appendicular skeletal muscle index (ASMI) was calculated as the ratio of appendicular muscle mass and squared height. The cutoff for low ASMI was less than 7.0 kg/m^2^ for men and less than 5.4 kg/m^2^ for women. Four participants who refused to undergo the scan were excluded from the whole analysis.

Lower limb strength was assessed by knee extension strength (KES) using the strap and strain gauge assembly component of the Physiological Profile Assessment described by Lord et al,^26^ and a mean value from 3 trials (standardized by sex and body mass index [BMI]) was calculated. Low KES was classified as less than or equal to 18 kg for men and less than or equal to 16 kg for women.

The 6-m fast gait speed (GS) test was performed as described by Nelson et al.^27^ Low GS was defined as a mean speed from 2 trials of less than or equal to 0.8 m/s.

A participant was categorized as having sarcopenia if he or she had both low ASMI and low KES and/or GS. Sarcopenia score was calculated as the number of positive components.

### Preintervention and Postintervention Assessments

At baseline, 3 months after intervention, and 6 months after intervention, participants underwent interviews and testing that included dual-energy x-ray absorptiometry scan, lower limb strength measurement, and 6-m fast GS test for the assessment and diagnosis of sarcopenia.

Weight and height were measured in light clothing, and BMI was calculated as weight in kilograms divided by height in meters squared. Cognitive function was measured using the Mini-Mental State Examination, which has been validated in local Singaporean elderly populations.^28^ The presence of depressive symptoms was determined by the 15-item Geriatric Depression Scale.^29^ Lung function was assessed using a spirometer to determine the ratio of forced expiratory volume in 1 second to the forced vital capacity predicted in the population of similar age, sex, and body composition.

### Statistical Analysis

The efficacy of interventions was examined using intention-to-treat analysis for clinical trial data. Group differences in means and proportions were compared by independent *t* test for continuous variables and χ^2^ test for categorical variables. The linear mixed model for the analysis of repeated measure data in longitudinal studies was used to investigate the effects of treatment group, time, and group × time interaction as fixed factors. For variables with significant group × time interaction indicating changing group effect over time, the simple main effect of treatment group was further evaluated at each point using 1-way analysis of variance with Bonferroni post hoc adjustments. Changes in sarcopenia and components at 3 months and 6 months after the intervention were compared between active intervention and standard care groups using multivariate linear models adjusting for baseline levels. Statistical significance was set at *P* < .05 using 2-tailed tests. All data analyses were performed using SPSS statistical software version 21 (IBM). Figures were generated using Prism graphing software version 7 (GraphPad).

## Results

### Baseline Characteristics and Functional Status of Study Participants With Sarcopenia

In 92 participants with sarcopenia, the mean (SD) age was 70.0 (4.7) years and 59 (64.1%) were female; all were of Chinese ethnicity. The 78 participants receiving active interventions and the 14 receiving standard care were comparable in demographic variables including mean age, sex, and formal education level (Table 1). Of 92 participants, 88 (95.6%) had low KES and 30 (32.6%) had low GS. No baseline difference was observed between the intervention group and the control group in terms of sarcopenia score and its components (ASMI, KES, and GS), BMI, daily time spent on physical activities, lung function, and mental health. Lower physical health score at baseline was observed in participants allocated to active interventions compared with those with standard care (*t* = 2.271; *P* = .03).

**Table 1.** Baseline Characteristics of Participants by Interventions Groups

Characteristic	Mean (SD)			Score, *t* or χ^2^	*P* Value
	All Participants (N = 92)	Intervention Groups			
		Active Interventions (n = 78)^a^	Standard Care (n = 14)		
Age, y	69.95 (4.72)	69.76 (4.31)	71.00 (6.65)	–0.675	.51
Female, No. (%)	59 (64.1)	53 (67.9)	6 (42.9)	3.249	.07
≥Secondary education, No. (%)	33 (35.9)	27 (34.6)	6 (42.9)	0.350	.55
Sarcopenia, No./total No. (%)	92/92 (100)	78/78 (100)	14/14 (100)		
Low appendicular skeletal muscle index, No./total No. (%)^b^	92/92 (100)	78/78 (100)	14/14 (100)		
Low knee strength, No./total No. (%)	88/92 (95.6)	75/78 (96.2)	13/14 (92.9)		
Low gait speed, No./total No. (%)	30/92 (32.6)	25/78 (32.1)	5/14 (35.7)		
Sarcopenia score^c^	2.28 (0.45)	2.28 (0.45)	2.29 (0.47)	–0.028	.98
Appendicular skeletal muscle index score^b^	5.32 (0.82)	5.25 (0.77)	5.69 (0.97)	–1.884	.06
Knee extension strength, kg	12.27 (3.11)	12.18 (3.11)	12.74 (3.22)	–0.621	.54
Gait speed, s	5.76 (1.76)	5.74 (1.78)	5.85 (1.74)	–0.212	.83
Frailty score^d^	2.20 (0.73)	2.19 (0.76)	2.21 (0.58)	–0.103	.92
Body mass index^e^	21.31 (2.55)	21.40 (2.62)	20.80 (2.16)	0.807	.42
Physical activity, min/d	158.20 (112.94)	159.11 (114.88)	153.14 (105.27)	0.181	.86
Lung function^f^	104.74 (19.19)	104.43 (19.71)	106.36 (16.80)	–0.342	.73
Mini-Mental State Examination score	29.11 (1.30)	29.18 (1.15)	28.71 (1.98)	0.855	.41
Geriatric Depression Scale score	0.67 (0.94)	0.68 (0.89)	0.64 (1.22)	0.134	.89
12-Item Short Form Survey					
Physical component score	49.67 (6.31)	49.24 (6.58)	52.11 (3.82)	–2.271	.03
Mental component score	54.37 (3.96)	54.63 (3.89)	52.94 (4.20)	1.476	.14

^a^ Active interventions included nutritional enrichment, cognitive training, physical exercise, and combined intervention.

^b^ Calculated as the ratio of appendicular muscle mass and squared height.

^c^ Calculated as the number of positive sarcopenia components.

^d^ Based on the criteria of unintentional weight loss, slow walking speed, weakness, self-reported exhaustion, and low physical activity.

^e^ Calculated as weight in kilograms divided by height in meters squared.

^f^ A spirometer was used to determine the ratio of forced expiratory volume in 1 second to the forced vital capacity predicted in the population of similar age, sex, and body composition.

### Baseline and Follow-up Results for Sarcopenia and Its Components

**Figure 2.**
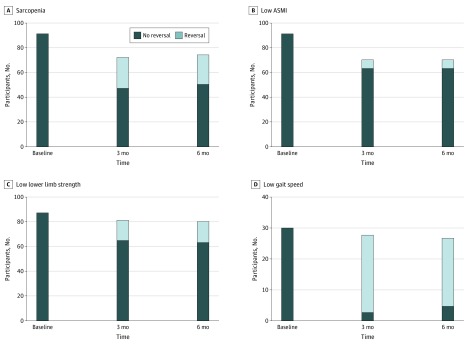
Association of Active Interventions With Sarcopenia and Component Functional Status Among 92 Participants at Baseline, 3 Months, and 6 Months Reversal was defined as the presence of sarcopenia at baseline and absence of sarcopenia or its components during follow-up. The components of sarcopenia included lower limb strength, appendicular skeletal muscle index (ASMI), and gait speed.

Of the 92 participants who had sarcopenia at baseline, the number who remained sarcopenic was reduced to 48 (of 73) after 3 months and 51 (of 75) after 6 months of intervention, indicating that 25 of 92 participants (27.2%) experienced sarcopenia reduction at 3 months and 24 of 92 (26.1%) had sarcopenia reduction at 6 months (Figure 2). Among the components of sarcopenia, GS had the greatest change associated with the active interventions: 25 of 30 participants (83%) were free of low GS at 3 months, and 22 of 30 (73.3%) were free of low GS at 6 months; in comparison, 16 of 88 participants (8.2%) were free of low KES at 3 months, 17 of 88 (19.3%) were free of low KES at 6 months, and 7 of 92 (7.6%) were free of low ASMI at 6 months (Figure 2).

As shown in Table 2, the mean (SD) sarcopenia score decreased from 2.28 (0.45) before the intervention to 1.80 (0.61) after the intervention in the active interventions group, indicating fewer positive sarcopenia components and improved sarcopenia status. The active intervention group experienced increases of ASMI from a mean (SD) of 5.25 (0.77) kg/m^2^ to 5.29 (0.75) kg/m^2^, increases in lower limb strength from a mean (SD) of 12.18 (3.11) kg to 14.24 (4.83) kg, and decreases in time to complete the 6-m gait speed test from a mean (SD) of 5.74 (1.78) seconds to 4.98 (1.02) seconds, suggesting improved muscle mass, lower limb strength, and gait speed after active interventions. Mixed-model analysis (Table 2) indicated a significant main effect of time for decreases of sarcopenia score (*F* = 14.138; *P* < .001) and time for gait speed test (*F* = 10.643; *P* < .001) at 3 months and 6 months of intervention. The effect for lower limb strength was not significant (*F* = 2.872; *P* = .06). There was a significant main effect of group for the improvement of ASMI (*F* = 9.627; *P* = .002). As shown in the eTable in the Supplement, the decreases in sarcopenia score and increases in ASMI, KES, and GS from baseline to 3 months and 6 months were statistically significant in the active intervention group. However, the differences between the active intervention group and the standard care (control) group were not statistically significant.

**Table 2.** Association of Multidomain Lifestyle Interventions With Sarcopenia and Its Components at 3 Months and 6 Months

Measurement	Mean (SD)		Time		Group		Time × Group	
	Interventions (n = 78)	Standard Care (n = 14)	*F *Score	*P* Value	*F *Score	*P* Value	*F *Score	*P* Value
Sarcopenia score								
Baseline	2.28 (0.45)	2.29 (0.47)	14.138	<.001	0.281	.60	0.121	.89
3 mo^a^	1.78 (0.62)	1.67 (0.49)						
6 mo^a^	1.80 (0.61)	1.75 (0.62)						
Appendicular skeletal muscle index, kg/m^2^								
Baseline	5.25 (0.77)	5.69 (0.97)	0.037	.96	9.627	.002	0.009	.99
3 mo	5.30 (0.74)	5.73 (0.64)						
6 mo	5.29 (0.75)	5.68 (0.79)						
Lower limb strength, kg								
Baseline	12.18 (3.11)	12.74 (3.22)	2.872	.06	0.252	.62	1.074	.34
3 mo^b^	13.70 (4.10)	15.18 (4.24)						
6 mo^c^	14.24 (4.83)	13.23 (4.07)						
Gait speed, s								
Baseline	5.74 (1.78)	5.85 (1.74)	10.643	<.001	0.745	.39	0.471	.63
3 mo^a^	4.76 (1.04)	4.45 (0.88)						
6 mo^a^	4.98 (1.02)	4.59 (0.99)						

^a^ *P* < .001 vs baseline level in post hoc pairwise comparisons that are significant.

^b^ *P* < .05 vs baseline level in post hoc pairwise comparisons that are significant.

^c^ *P* < .01 vs baseline level in post hoc pairwise comparisons that are significant.

### Baseline Demographic and Physical Characteristics of Participants With Sarcopenia Reduction

**Figure 3.**
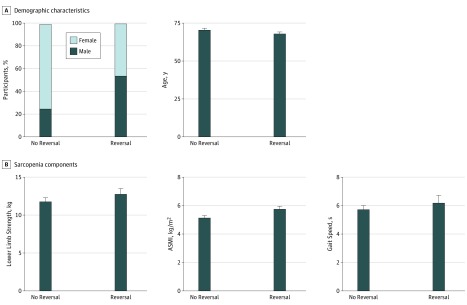
Characterization of Demographic Characteristics and Sarcopenia Components of Participants With Sarcopenia Reversal A, Demographic characteristics for the 24 participants who experienced sarcopenia reversal (defined as the presence of sarcopenia at baseline and absence of sarcopenia at 6 months) and the 51 participants who experienced no reversal (defined as the presence of sarcopenia at both baseline and 6 months). Differences were statistically significant for both sex and mean age, with male participants and those who were younger experiencing greater rates of reversal. B, Reversal of individual sarcopenia components. Participants whose sarcopenia was reduced at 6 months had significantly higher baseline appendicular skeletal muscle index (ASMI) levels than those who remained sarcopenic at 6 months. Error bars represent standard error.

Baseline demographic and physical characteristics varied between the 24 participants who experienced a reduction in sarcopenia at 6 months and the 51 who did not. There was a much higher proportion of male participants in the group with reduction (54.2%) than in the group with no reduction (25.5%) (χ^2^ = 5.925, *P* = .02). Patients who experienced reduction were also younger (mean [SD] age, 68.42 [3.37] years vs 70.88 [5.32] years; *t* = −2.078; *P* = .04) (Figure 3A). Participants whose sarcopenia was reduced at 6 months had significantly higher baseline ASMI levels than those who remained sarcopenic at 6 months (mean [SD] ASMI, 5.74 [0.77] vs 5.14 [0.77] kg/m^2^; *P* = .002). There was no statistically significant difference in baseline lower limb strength (mean [SD], 12.88 [3.14] vs 11.88 [2.99] kg; *P* = .19) and gait speed (mean [SD], 6.17 [2.37] vs 5.71 [1.55] seconds; *P* = .40) between the group that experienced reduction in sarcopenia and the group that did not (Figure 3B).

## Discussion

A limited number of studies have previously examined the association of physical exercise and/or nutritional interventions with sarcopenia reduction.^15,16,17,18,19^ Most of these earlier studies defined sarcopenia based solely on a low skeletal muscle mass index; only 1 study^15^ additionally assessed muscle function. In this study, we observed that community-dwelling prefrail and frail older persons with sarcopenia who were participants in multidomain lifestyle interventions demonstrated reduced sarcopenia and increased muscle mass and function at 3 months and 6 months, with one-third of the participants with sarcopenia at baseline having no sarcopenia after the intervention. Notably, low GS was present in only one-third of the participants with sarcopenia, but showed by far the most pronounced improvement compared with muscle mass or strength. There was a pronounced placebo effect in the usual care control group (which was transiently observed at 3 months), possibly explainable by the novel exposure to active participation in a trial among these usually inactive prefrail and frail individuals. Because of this, there was a lack of statistically significant difference vs the usual care group. This contrasts with our previous observations^22^ of the clearly positive effect of active interventions over usual care in the larger trial group of prefrail and frail participants, which included individuals without sarcopenia. The results nonetheless suggest that older persons with sarcopenia may respond to active multidomain lifestyle interventions, as the absolute effect size of 2.2-kg gain in muscle strength at 6 months is comparable to that observed in the previous study.^22^ The estimated gain of 0.6 kg in muscle strength at 6 months in the usual care control group was larger than that observed (0 kg) in the other, larger, study. Sampling error in this estimate due to the small number of participants in the usual care control group may explain this result.

Notably, the randomized clinical trial^22^ applied moderate and gradually increasing intensity of physical exercise tailored to the needs and tolerance of the participants and achieved a high compliance rate (85%). The observed improvements in lower limb strength and gait speed after physical exercise are consistent with findings from other studies.^15,16,30,31^ There are studies suggesting that high-intensity exercise training is especially associated with improvement of muscle strength,^32^ but high-intensity exercise is difficult to implement in sarcopenic elderly populations. In line with previous studies that failed to demonstrate robust positive association of nutritional intervention with the improvement of sarcopenia^15,17,33^ or physical functions^34,35^ in older persons, we had also reported^22^ a lack of association of nutritional intervention with improvement of muscle functions. However, nutritional enrichment combined with physical exercise and cognitive training was strongly associated with increased muscle strength.

Sarcopenia reduction from active interventions appeared to favor those who were male, were younger, and had greater muscle mass. These findings may be useful in informing interventional initiatives to reduce sarcopenia. The characterization of those who might benefit most from the interventions facilitates the identification of target populations in future sarcopenia trials.

### Limitations

This study has limitations. The generalization of the results to all elderly populations should be cautioned considering the unique features of this study. The high compliance rates to the intervention programs achieved via excellent rapport with the participants may be exceptional to this randomized clinical study. The participants who were excluded from participation in the trial at prescreening were predominantly very frail and functionally disabled. The interventional responses among these study participants who are community-dwelling Chinese prefrail and frail elderly individuals without cognitive impairment or frequent hospitalization may differ from interventions for hospitalized or institutionalized elderly people. Given that this study is a secondary post hoc analysis of data in a subset of participants with sarcopenia in a randomized clinical trial of prefrail and frail older persons, the current findings are tentative and warrant further investigation.

## Conclusions

In conclusion, aging is associated with the steady dramatic decline of lean mass and associated physical function. This post hoc analysis of a community-based randomized clinical trial demonstrated associations between multidomain lifestyle interventions (physical, nutritional, and cognitive interventions) among prefrail and frail older persons with reductions in sarcopenia, especially in those who were male, were younger, or had larger muscle mass. Slow gait, present in a third of older persons with sarcopenia, had the strongest association with active lifestyle interventions.

## Notes

Supplement. **eTable.** Changes in Sarcopenia and Components at 3 Month and 6 Month by Intervention and Standard Care Groups
