# Supplementary material for: Assessment of Sarcopenia Among Community-Dwelling At-Risk Frail Adults Aged 65 Years and Older Who Received Multidomain Lifestyle Interventions: A Secondary Analysis of a Randomized Clinical Trial
Source: JAMA Netw Open. 2019 Oct 16;2(10):e1913346. doi: 10.1001/jamanetworkopen.2019.13346 (PMC6806429; doi:10.1001/jamanetworkopen.2019.13346)
Supplement: Supplement. — eTable. Changes in Sarcopenia and Components at 3 Month and 6 Month by Intervention and Standard Care Groups [file jamanetwopen-2-e1913346-s001.pdf]

Supplementary Online Content

Lu Y, Niti M, Yap KB, et al. Assessment of sarcopenia among community-dwelling at-risk frail adults aged 65 years and older who received multidomain lifestyle interventions: a secondary analysis of a randomized clinical trial. *JAMA Netw Open*. 2019;2(10):e1913346. doi:10.1001/jamanetworkopen.2019.13346

eTable. Changes in Sarcopenia and Components at 3 Month and 6 Month by Intervention and Standard Care Groups

This supplementary material has been provided by the authors to give readers additional information about their work.

© 2019 Lu Y et al. *JAMA Network Open.*

**eTable. Changes in sarcopenia and components at 3 month and 6 month by intervention and standard care groups**				
**Changes (Follow up – baseline)**<br>(mean (95% CI)	**Interventions**<br>(n = 78)	**Standard Care**<br>(n = 14)	t/ F	*p*
**Sarcopenia score** (mean (95% CI))				
3 Month	-0.53 (-0.71, -0.34)*	-0.58 (-1.09, -0.08)*	0.254	0.800
6 Month	-0.51 (-0.69, -0.33)*	-0.50 (-1.01, 0.01)	-0.037	0.970
Adjusted (baseline level)				
3 Month	-0.52 (-0.67, -0.36)*	-0.62 (-0.97, -0.28)*	0.308	0.581
6 Month	-0.50 (-0.66, -0.34)*	-0.54 (-0.89, -0.19)*	0.038	0.847
**ASMI, kg/m^2** (mean (95% CI))				
3 Month	0.10 (0.03, 0.17)*	0.08 (-0.27, 0.42)	0.237	0.813
6 Month	0.08 (0.02, 0.15)*	0.03 (-0.34, 0.39)	0.347	0.735
Adjusted (baseline level)				
3 Month	0.09 (0.01, 0.17)*	0.13 (-0.04, 0.31)	0.220	0.641
6 Month	0.08 (-0.01, 0.16)	0.07 (-0.12, 0.26)	0.001	0.971
**Lower limb strength, kg** (mean (95% CI))				
3 Month	1.52 (0.56, 2.47)*	2.54 (-0.31, 5.39)	-0.812	0.419
6 Month	2.22 (1.19, 3.24)*	0.59 (-2.30, 3.49)	1.227	0.223
Adjusted (baseline level)				
3 Month	1.48 (0.58, 2.38)*	2.75 (0.61, 4.88)*	1.180	0.280
6 Month	2.19 (1.17, 3.20)*	0.76 (-1.62, 3.15)	1.191	0.278
**Gait speed, seconds** (mean (95% CI))				
3 Month	-0.97 (-1.39, -0.56)*	-1.33 (-2.40, -0.26)*	0.666	0.507
6 Month	-0.78 (-1.20, -0.35)*	-1.19 (-2.37, -0.02)*	0.757	0.451
Adjusted (baseline level)				
3 Month	-0.98 (-1.21, -0.76)*	-1.30 (-1.83, -0.76)*	1.186	0.279
6 Month	-0.78 (-1.01, -0.55)*	-1.18 (-1.72, -0.64)*	1.781	0.186

ASMI = Appendicular skeletal muscle index. Figures in parentheses are 95% confidence intervals.
*indicates estimates of changes from baseline that are statistically significant at $p < 0.01$.

© 2019 Lu Y et al. *JAMA Network Open.*
